# Porous VO_x_N_y_ nanoribbons supported on CNTs as efficient and stable non-noble electrocatalysts for the oxygen reduction reaction

**DOI:** 10.1038/srep17385

**Published:** 2015-11-30

**Authors:** K. Huang, K. Bi, Y. K. Lu, R. Zhang, J. Liu, W. J. Wang, H. L. Tang, Y. G. Wang, M. Lei

**Affiliations:** 1 State Key Laboratory of Information Photonics and Optical Communications & School of Science, Beijing University of Posts and Telecommunications, Beijing 100876, China; 2 School of Materials Science and Engineering, Central South University, Changsha, Hunan, 410083, China; 3 Beijing National Laboratory for Condensed Matter Physics, Institute of Physics, Chinese Academy of Sciences, Beijing 100190, China; 4 State Key Laboratory of Advanced Technology for Materials Synthesis and Processing, Wuhan University of Technology, Wuhan 430070, China

## Abstract

Novel nanocomposites of carbon nanotubes supported porous VO_x_N_y_ nonoribbons (VO_x_N_y_-CNTs) have been synthesized by the annealing of the sol-gel mixture of CNTs and V_2_O_5_ under NH_3_ atmosphere as well as the ageing process in air. Besides the morphological and structural characterizations revealed by TEM, SEAD, EDS, XRD and XPS measurements, typical electrochemical tests including cyclic voltammetry (CV), rotating disk electrode (RDE) and chronoamperometry have been employed to determine the oxygen reduction reaction (ORR) performance of VO_x_N_y_-CNTs. Inspiringly, the results indicate that VO_x_N_y_-CNTs catalyst exhibits a 0.4 mA/cm^2^ larger diffusion-limited current density, a 0.10  V smaller onset potential value, a 10.73% less of ORR current decay and an excellent methanol-tolerance compared with commercial Pt/C catalyst. Therefore, we have reasonable grounds to believe that this new VO_x_N_y_-CNTs nanocomposites can be regarded as a promising non-precious methanol-tolerant ORR catalyst candidate for alkaline fuel cells.

With the highly social development of industrialization and urbanization, the critical energy shortages mainly from the depletion of fossil fuels have motivated and accelerated intensive researches in developing alternative energy conversion and storage devices (ECSDs) with higher power and energy densities^1^,^2^,^3^,^4^,^5^. Among various potential ECSDs for practical applications, polymer electrolyte membrane fuel cells (PEMFCs) have been extensively regarded as ideal power sources for future mobile and stationary applications due to their high energy efficiency, high power density and low level of emissions^6^,^7^,^8^. As important as the biological respiration in life processes, the oxygen reduction reaction (ORR) as the key cathodic process in PEMFCs will determine the behavior of the whole device due to the sluggish kinetics with respect to the anodic electrochemical reactions^9^. Thus, Pt-dominated materials have been used as highly desired catalysts to accelerate the ORR process in order to obtain an enhanced overall performance of PEMFCs. However, the elevated price and extreme CO or methanol sensitivities of the scarce platinum element have limited their widespread use in practical applications, and therefore developing non-precious ORR catalysts with efficient activity and stability has increasingly become the key issue for the commercialization of fuel cells.

Meanwhile, transition metal nitrides and oxynitrides have been intensively researched as suitable electrode or catalyst materials in recent years for various ECSDs, including fuel cells, metal-air batteries, lithium-ion batteries (LIBs), dye-sensitized solar cells (DSSCs) and electrochemical capacitors (ECs)^10^,^11^,^12^,^13^,^14^,^15^,^16^, due to the unique physical and chemical properties such as high intrinsic conductivity, relatively good electrochemical stability as well as the Pt-like electrocatalytic activity. And more especially, great interests have also been aroused in the development of vanadium nitrides and oxynitrides as advanced electrode materials by the nitridation of various vanadium oxides precursors. For example, Zhou *et al*. synthesized VN powder by calcining V_2_O_5_ xerogel under NH_3_ atmosphere with a promising supercapacitive specific capacitance of 161 F/g in 1M KOH at 30 mV/s^17^. Glushenkov prepared porous nanocrystalline VN by temperature-programmed ammonia reduction of V_2_O_5_ powder which exhibited a capacitance of 186 F/g in 1M KOH electrolyte at a current load of 1 A/g^18^. Porto’s group investigated the capacitances of VO_x_N_y_ obtained from the nitridation of commercial V_2_O_5_ and VO_2_ precursors with the best specific capacitance of 186 F/g in 1M KOH at 10 mV/s^19^. To further enhance the electrochemical properties of VO_x_N_y_ powders, Chen and his coworker synthesized carbon-supported VO_x_N_y_ nanocrystalline using a mixture of melamine and V_2_O_5_ xerogel as precursor and obtained a specific capacitance of 273 F/g in 1M KOH at the scan rate of 30 mV/s^20^. Shu *et al*. also developed a soft-template synthesis of VO_x_N_y_-C nanomaterials for supercapacitors with a maximum specific capacitance of 271 F/g at 1A/g using ployvinylpyrrolidone (PVP) as the template and V_2_O_5_ xerogel as vanadium source. Their results demonstrated that the intimate contact between VO_x_N_y_ grains and remaining carbon will result in a better electronic conductivity and the larger surface area will furnish more surface active redox sites^21^.

Although the occurrence of fast faradic redox reactions as well as the formation of electrical double-layer on the surface of VN and VO_x_N_y_ have been suggested as the supercapacitive mechanism, few works have yet employed vanadium nitrides and oxynitrides as possible non-precious catalysts towards ORR. Huang *et al*. developed a hydrothermal route followed by calcination to prepare VN powder, which exhibited a comparable diffusion-limited current density but an inferior onset potential to commercial Pt/C catalyst^22^. Our previous work about VN/C catalyst also indicated the same tendency with the addition of the superior stability and methanol-tolerance^23^. Other than loading with Pt particles to obtain an enhanced ORR performance as shown in Yin’s work^24^, the introduction of porous structure and high surface area supporter can also elevate their electrochemical behaviors due to the increased conductivity and number of active sites^9^,^25^. Currently, the development of novel multifunctional composites/hybrids by structuring porous materials into the other nanostructures has been regarded as a rapidly developing research area. It is because that the composites/hybrids usually exhibit new properties that are superior to those of the individual components due to the collective behavior of the functional units. Thus, these emerging composites/hybrids will stimulate the emergence of innovative industrial applications in various important fields, including functional and protective coatings, storage and separation, heterogeneous catalysis, sensing and biology^26^,^27^. Herein, we have successfully synthesized novel CNTs-supported porous VO_x_N_y_ nanoribbons composites with an improvement of structural features as efficient and durable non-precious ORR catalysts in alkaline electrolyte using V_2_O_5_ nanoribbons and CNTs as raw materials.

## Results

**Figure 1:**
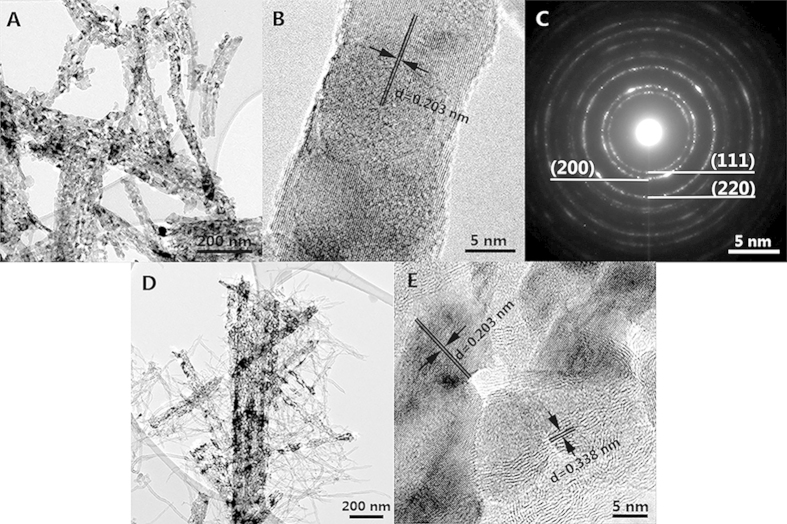
TEM images at different magnifications of VO_x_N_y_ (A and B) and VO_x_N_y_-CNTs (D and E); SAED image (C) of VO_x_N_y_-CNTs.

**Figure 2:**
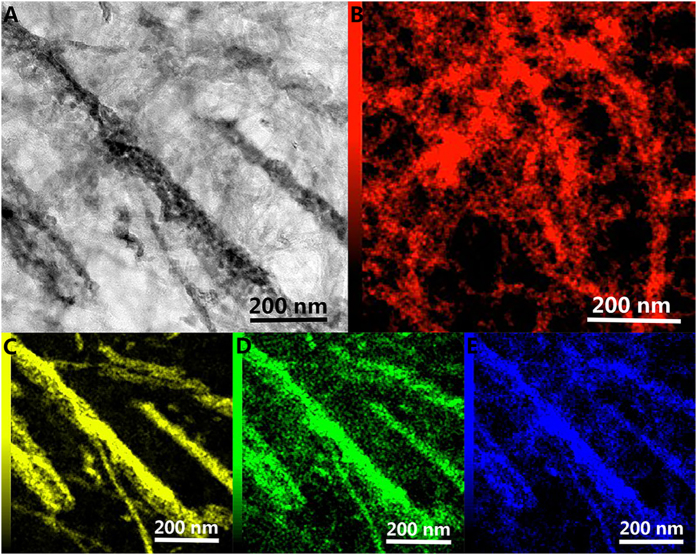
TEM image (A) and element mapping spectra about C (B), V (C), O (D) and N (E) of VO_x_N_y_-CNTs.

**Figure 3:**
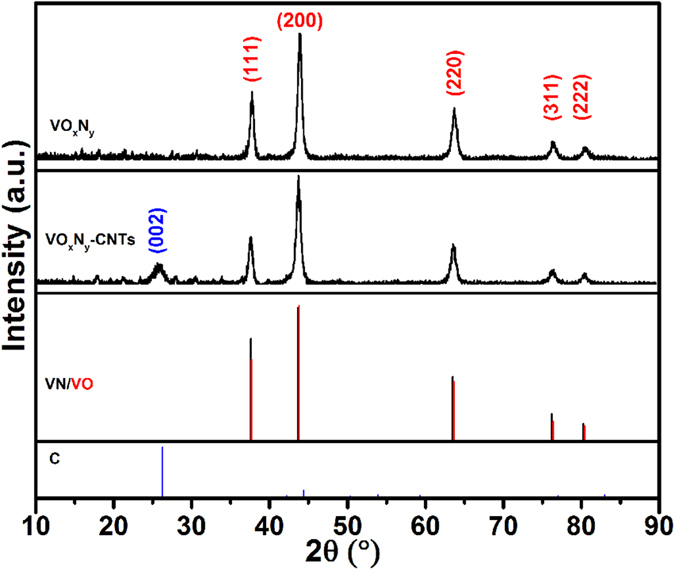
XRD Patterns of VO_x_N_y_ and VO_x_N_y_-CNTs with the standard patterns of VN, VO and graphite carbon.

Figure 1 shows the morphological characters of as-prepared samples by transmission electron microscope (TEM) equipped with the selected area electron diffraction (SAED). It can be seen that there are plentiful pores distributing in the VO_x_N_y_ nanoribbons due to the topotactic transformation whether supported by CNTs or not (Fig. 1A,D). The high-resolution TEM images show clear lattice fringes with an interfringe spacing of 0.203 nm which corresponds to the d-spacing of (200) planes (Fig. 1C). As for VO_x_N_y_-CNTs composites, VO_x_N_y_ nanoribbons with a few tens of nanometers in widths and lengths up to micrometer range are randomly supported by plentiful tortuous CNTs (which are more transparent to the electron beam). Moreover, the element mapping spectra visually displayed in Fig. 2 demonstrate the locations of VO_x_N_y_ nanoribbons in the composites. Figure 2B shows the C element from CNTs supporter which distributes everywhere and randomly, while the distributions of V, O and N elements are in accordance with the VO_x_N_y_ nanoribbons to a great extent and the messy existence of O and N elements in Fig. 2C,D is believed to come from the surface species of CNTs. As the XRD patterns shown in Fig. 3, five main peaks ranging from 35° to 85° for both VO_x_N_y_ and VO_x_N_y_-CNTs can match well with (111), (200), (220), (311) and (222) planes of the typical stoichiometric face-centered (fcc) VO (JCPDS No. 75-0048) or VN (JCPDS No. 73–0528) structures, where the figure of merit (FOM) values are 1.2 and 5.6 respectively. This result can be ascribed to the penetration of oxygen atoms into the VN crystal lattice^17^, which happened once the prepared samples were taken off from the furnace. Moreover, the broad peak for VO_x_N_y_-CNTs sample appears at about 26° which corresponds to the (002) plane of graphite carbon (JCPDS No. 75–1261) and confirms the existence of CNTs.

**Figure 4:**
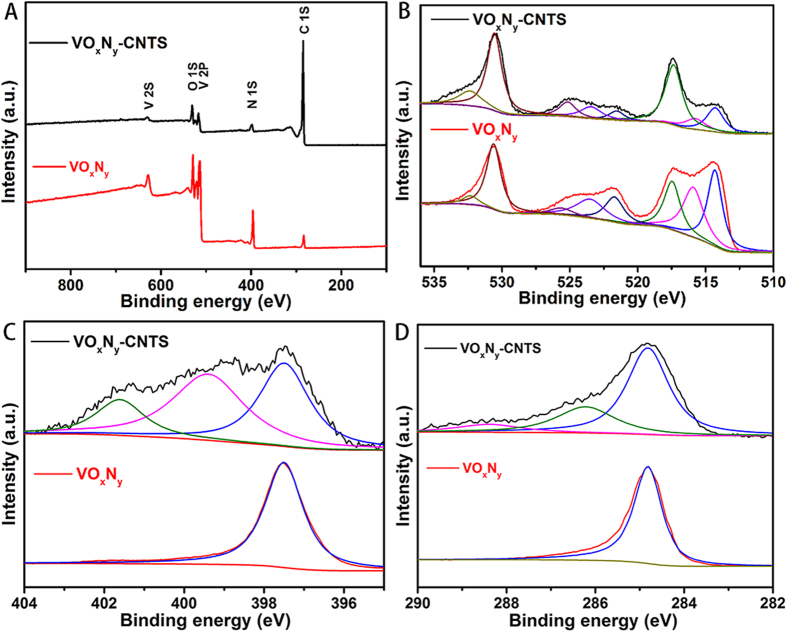
XPS spectra of VO_x_N_y_ and VO_x_N_y_-CNTs: survey (A), O 1 s and V 2P (B), N 1S (C) and C 1S (D).

XPS analysis in Fig. 4 further reveals that the components of VO_x_N_y_ and VO_x_N_y_-CNTs are consist of V, N, O, and C elements from the survey spectra. Compared with the high intensity peak of CNTs in VO_x_N_y_-CNTs nanocomposites, it is notable that the signal of C 1S peak for pure VO_x_N_y_ nanoribbons can be attributed to the air contamination on surface^28^, which exhibits a symmetric peak at 284.8 eV (Fig. 4D). Figure 4B shows the high-resolution XPS spectra of O 1 s and V 2p, where two peaks at 514.2 V (2p 3/2) and 521.5 eV (2p 1/2) can be ascribed to vanadium atoms in the VN crystalline, other four vanadium-based peaks at 515.7, 517.3, 523.4 and 525.1 eV are belong to vanadium oxides and the peaks at 532.2 and 530.3 eV of O 1s can be attributed to lattice oxygen in V_x_O_y_ due to the ageing process in air and surface adsorbed hydroxyl oxygen, respectively^29^,^30^,^31^,^32^,^33^. In addition, it is found that the chemical states of N and C elements for VO_x_N_y_-CNTs are quite different from pure VO_x_N_y_ nanoribbons as shown in Fig. 4C,D. The peak of N 1s spectra at 397.5 eV can be assigned to the corresponding metal nitrides while the other two peaks at 399.5 and 401.3 eV are belonging to pyrrolic and quaternary-graphitic separately^31^. The C 1S spectrum also exhibits a typical aromatic C-N coordination in a graphitic carbon nitride framework at 288.2 eV except for the non-oxygenated ring C (284.8 eV, C-C, C = C) as well as the C-O bond at 286.3 eV^34^,^35^. These results indicate that a surface modification and N-doping of CNTs happened sequentially during the acid treatment and annealing process, which have been proved to show excellent ORR activity^36^,^37^,^38^.

**Figure 5:**
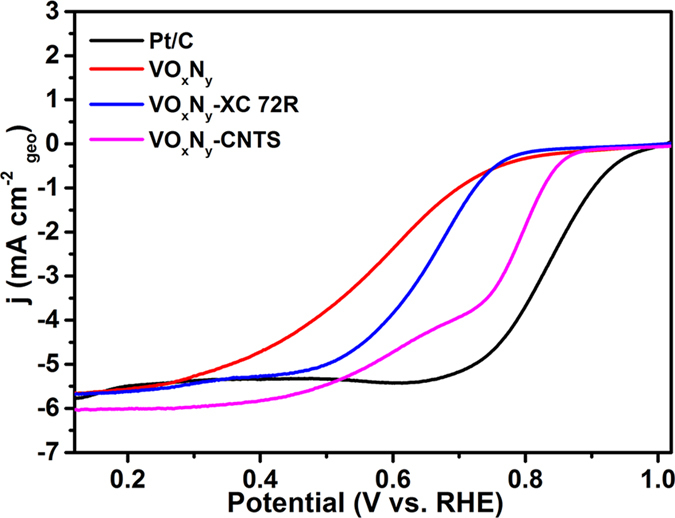
ORR activities of different VO_x_N_y_-based catalysts compared with Pt/C catalyst at 1600 rpm.

**Figure 6:**
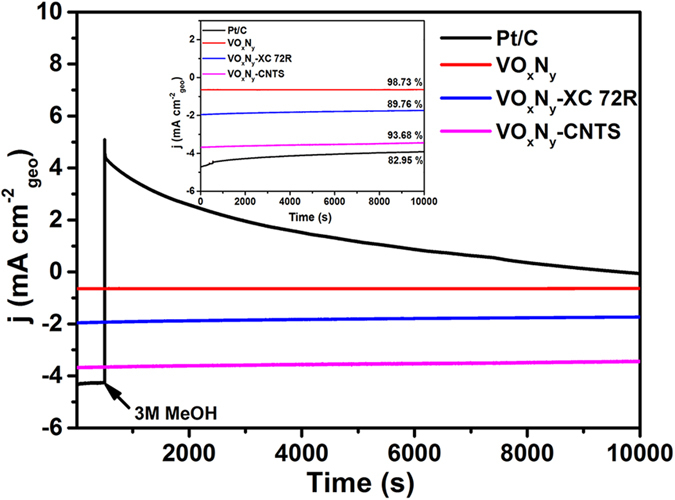
The chronoamperometric responses of different VO_x_N_y_-based electrodes as well as commercial Pt/C electrode with or without (insert) adding 3M MeOH at 500 s.

The ORR performances of VO_x_N_y_ and VO_x_N_y_-CNTs catalysts with VO_x_N_y_-XC 72R and commercial Pt/C as the references have been further investigated in alkaline electrolyte. As shown in Fig. 5, the ORR polarization curves for all kinds of catalysts at 1600 rpm in O_2_-saturated 0.1 M KOH exhibit a combined kinetic-diffusion control of charge transfer and mass transport process at different potentials. It is obvious that the support of carbon can improve the onset potential, half-wave potential and the diffusion-limited current density of pure VO_x_N_y_ nanoribbons catalyst, and the hybrid with CNTs displays the strongest competitiveness with respect to the commercial Pt/C. Although the onset potential (0.88 V Vs. RHE) and half-wave potential (0.77 V Vs. RHE) of VO_x_N_y_-CNTs are 0.10 and 0.07 V smaller than those of Pt/C electrode, the diffusion-limited current density is about 0.4 mA/cm^2^ larger. Meanwhile, the stabilities of VO_x_N_y_-based electrodes are quite superior to that of Pt/C electrode no matter whether adding 3M Methanol at 500 s. As shown in the inset of Fig. 6, the current densities of pure VO_x_N_y_ electrode, VO_x_N_y_-Xc 72R electrode, VO_x_N_y_-CNTs electrode and Pt/C electrode remain 98.73%, 89.76%, 93.68% and 82.95% after 1000 s compared with the initial ones. The increased loss with the addition of XC 72R and CNTs can be attributed to the oxidation of carbon under dynamic potential conditions and high-oxygen environment^39^,^40^. Furthermore, unlike the instantaneous current density jump for Pt/C electrode due to the addition and the following electro-oxidation of methanol^41^, all VO_x_N_y_-based electrodes exhibit superior methanol tolerance with negligible changes. In addition, the ORR performance in alkaline electrolyte of VO_x_N_y_-CNTs is also highly comparable to other widely studied non-precious electrocatalysts, such as 3D nitrogen-doped graphene aerogel-supported Fe_3_O_4_ nanoparticles (Fe_3_O_4_/N-GAs)^42^, carbon nanotubes supported MnO_x_ hybrids (MnO_x_/CNTs)^43^, carbon-supported CoSe_2_ nanocatalyst (CoSe_2_/C)^44^, cobalt and nitrogen-cofunctionalized graphene (Co-N-GN)^45^ and Fe_2_N-N-doped graphitic nanocarbons composite (Fe_2_N-NGC)^46^.

## Discussion

**Figure 7:**
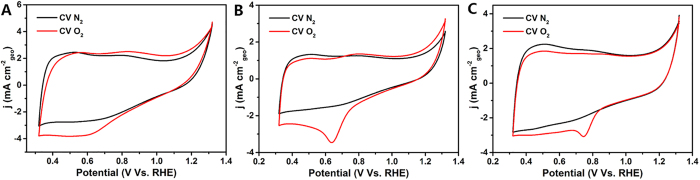
CV curves of VO_x_N_y_ (A), VO_x_N_y_-XC 72R (B) and VO_x_N_y_-CNTs (C) as ORR catalysts in N_2_/O_2_-saturated 0.1 M KOH solution.

To further understand the enhanced performance of VO_x_N_y_-CNTs catalysts with respect to VO_x_N_y_-XC 72R and pure VO_x_N_y_ electrodes, cyclic voltammetry (CV) curves under both N_2_- and O_2_-saturated 0.1 M KOH electrolyte have been also recorded in Fig. 7. As can be seen, there are no obvious oxidation or reduction current peaks in all CV curves under N_2_-saturated KOH solution differing from Fe-N/C catalysts which present redox couples due to the existence of instable metal ions^47^. Thus, this result also indicates the high electrochemical stability of VO_x_N_y_ over the whole measured potential range. Meanwhile, significant ORR peaks can be observed for VO_x_N_y_-XC 72R (0.65 V vs. RHE) and VO_x_N_y_-CNTs (0.75 V vs. RHE) electrodes under the O_2_-saturated condition compared with pure VO_x_N_y_ electrode, which suggests the decreased overpotential and favorable charge transfer process for ORR^48^,^49^. Considering the above results of ORR polarization curves and chronoamperometric responses, VO_x_N_y_-CNTs composites exhibit a fascinating ORR activity and stability. As is well-known to all, ORR is a surface chemical process where the consumption of oxygen occurs on the surface of electrode materials. Therefore, the enhanced performance of VO_x_N_y_-CNTs composites is believed to be closely related to the surface chemical states: (i) the bond between the carbon and the nitrogen is favorable to elevate the ORR electro-catalytic activity; (ii) the surface VO_x_N_y_ layer itself benefits the stability and tolerance of methanol.

**Figure 8:**
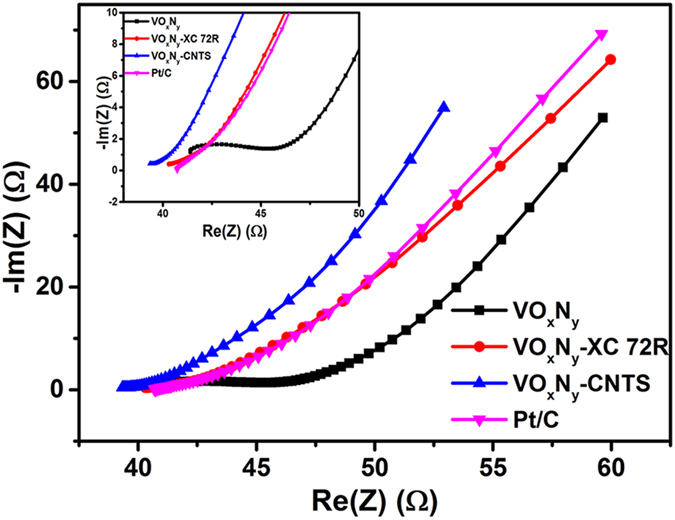
Nyquist plots corresponding to all referred electrodes in 0.1 M KOH solution from 100 kHz-1 Hz; The inset shows the low impedance region.

On the other hand, the increased overall conductivity also contributes to the decrease of overpotential as demonstrated before^50^. To further monitor the different conductivity of Pt/C and all VO_x_N_y_-based electrodes, representative Nyquist plots of electrochemical impedance spectroscopy (EIS) are displayed in Fig. 8, where the high frequency region (see the inset) can be associated with the charge-transfer process as well as the properties of electrochemical reaction resistance and the low frequency straight lines relate to the properties of the diffusion process^51^. It is obvious that the initial resistances corresponding to the combination of solution resistance and the film resistance are 39.3, 40.2, 40.6 and 41.4 Ω for VO_x_N_y_-CNTs, VO_x_N_y_-XC 72R, commercial Pt/C and bare VO_x_N_y_ electrodes respectively, which indicates the enhanced electrochemical conductivity due to the introduction of carbon support for VO_x_N_y_ catalyst. Moreover, the apparent arc-shaped region for just bare VO_x_N_y_ electrode which is relative to the charge-transfer resistance is also responsible for its large ORR overpotential. Thus, the highest overall conductivity of VO_x_N_y_-CNTs is beneficial to the higher ORR onset potential, i.e., the lower overpotential.

**Figure 9:**
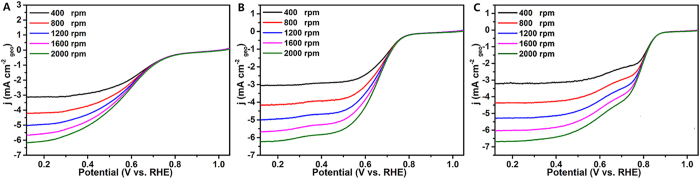
ORR polarization curves of VO_x_N_y_ (A), VO_x_N_y_-XC 72R (B) and VO_x_N_y_-CNTs (C) as electrocatalysts in O_2_-saturated 0.1 M KOH solution.

**Figure 10:**
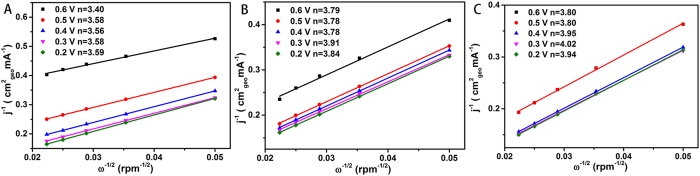
Koutechy-Levich (K-L) plots at different potentials for VO_x_N_y_ (A), VO_x_N_y_-XC 72R (B) and VO_x_N_y_-CNTs (C) electrodes.

Furthermore, since the ORR is a multi-electron charge transfer reaction with two main possible paths in alkaline electrolyte: one is one step direct pathway, involving four electrons transfer to produce 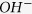
while the other one is two steps indirect pathway, involving two electrons transfer to produce 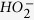 and then the 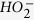 get another two electrons to transform into 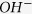, rotating disk electrode (RDE) voltammetry (i.e. linear-sweep voltammetry, LSV) was performed in oxygen-saturated 0.1 M KOH solution at a scan rate of 5 mV/s with the rotation rate from 400 to 2000 rpm to gain further insight into the ORR process. Figure 9 shows that all VO_x_N_y_-based electrodes display elevated ORR current densities with the increase of rotation rate and VOxNy-CNTs perform best in view of all above three electrochemical parameters. In addition, the numbers of electrons transferred per oxygen molecule in ORR have been estimated on the basis of the well-known Koutechy-Levich (K-L) equation:


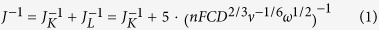


where *J* and *J*_*K*_ are the measured and kinetic current density, 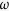 is the rotation rate in the form of rpm, 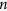 is the transferred electron number, 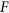 is the Faraday constant (96485 C mol^−1^), 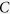 is the concentration of O_2_ (1.2
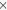
10^−6^ mol cm^−3^), 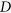 is the diffusion coefficient of O_2_ (1.9
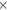
10^−5^ cm^2^ s^−1^), 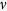 is the kinematic viscosity (0.01 cm^2^ s^−1^) in 0.1 M KOH solution^52^. As shown in Fig 10, the electron transfer numbers of VO_x_N_y_, VO_x_N_y_-XC 72R and VO_x_N_y_-CNTs electrodes at all selected potentials increase in sequence with the average electron transfer numbers of 3.54, 3.82 and 3.90 respectively. Thus, this results indicate that VO_x_N_y_-CNTs catalyst exhibits a direct 4-electron dominated reduction process. And consistent with the analysis of EIS measurements, the increased number of electron transferred also suggests that the addition of carbon support can facilitate the transfer of electron from the surface of catalysts to oxygen molecule.

In conclusion, we have successfully prepared novel porous VO_x_N_y_-CNTs nonocomposite which exhibits tremendous potential to be used as an appropriate non-noble ORR electrocatalyst with superior stability, excellent methanol-tolerance and competitive activity. Combining the results of electrochemical tests and structural characterizations, the porous structure among VO_x_N_y_ nanoribbons can help the electrolyte and oxygen make adequate contact with the catalysts, the presence of CNTs as well as the formation of carbon-nitrogen bonds can further enhance the overall conductivity and reduce the overpotential for the motivation of ORR while the surface VO_x_N_y_ layer itself benefits the tolerance of methanol.

## Methods

### Synthesis of VO_x_N_y_-CNTs nanocomposite

V_2_O_5_ nanobelts using as precursor were first prepared by a typical hydrothermal process, in which 2 mmol V_2_O_5_ powder was added into a mixture of 30 mL DI water and 5 mL H_2_O_2_ (30%) under vigorous stirring for 30 min to obtain a clear dark red solution before being transferred into a 50 mL of Teflon-sealed autoclave at 200 °C for four days. Meanwhile, 0.5 g of home-made CNTs (8–15 nm in diameter and 0.5–2 μm in length) were added into 50 mL of 98% H_2_SO_4_ and 14 mL of 65% HNO_3_ solution, refluxed at 80 °C for 90 min and then recovered by centrifugation. Then, V_2_O_5_ nanobelts and acid-treated CNTs (V_2_O_5_: CNTs = 6: 4) were mixed together uniformly in certain DI water with the aids of ultrasonication and stirring. After the vacuum filtration, peeling off from the membrane and drying treatment, the obtained mixture powder was further annealed in NH_3_ at 600 °C for 1 h with a ramping rate of 5 °C/min. Subsequently, the as-prepared sample was cooled down to room temperature under high purity nitrogen atmosphere. Moreover, pure VO_x_N_y_ sample was obtained under the same conditions without the addition of CNTs.

### Structure analysis of the VO_x_N_y_-based samples

Morphology and microstructure of the samples were characterized by powder X-ray diffractometry (XRD, X’pert PRO, Panalytical) using Cu Kα as radiation source, transmission electron microscopy (TEM, ARM200F, JEM) equipped with energy-dispersive X-ray spectroscopy (EDS). XPS measurements were carried out on an ESCALAB 250Xi spectrometer by Thermo Scientific with C 1s peak at 284.8 eV as an internal standard.

### Electrochemical measurement of the VO_x_N_y_-based electrodes

All of the electroch-emical tests were performed on a three-electrode system (a glassy carbon electrode of 5 mm in diameter with a catalyst loading of 0.5 mg cm^−2^ as the working electrode, a Pt foil as the counter electrode and an Hg/HgO electrode as the reference electrode) using Autolab PGSTAT-204 and CHI 660E workstations. All potential values were calibrated with respect to reversible hydrogen electrode by E_RHE_ = E_Hg/HgO_ + 0.92 V. What’s more, the preparation of VO_x_N_y_, VO_x_N_y_-XC 72R (60% VO_x_N_y_) and VO_x_N_y_-CNTs electrodes are similar to our previous works^23^,^53^. Cyclic voltammetry (CV) measurements were performed at a scan rate of 50 mv s^−1^ in N_2_-/O_2_-saturated 0.1 M KOH electrolyte. Rotating disk electrode (RDE) tests were conducted at different rotating speeds from 400 to 2000 rpm in O_2_-saturated 0.1M KOH solution at a scan rate of 5 mv s^−1^. The chronoamperometry responses (i-t curves) were carried on a constant voltage of 0.65 V in O_2_-saturated 0.1 M KOH solution with or without adding 3M methanol at 500 s. Electrochemical impedance spectra (EIS) were measured from 1 Hz to 100 kHz with a potential amplitude of 10 mV.

## Additional Information

**How to cite this article**: Huang, K. *et al*. Porous VO_x_N_y_ nanoribbons supported on CNTs as efficient and stable non-noble electrocatalysts for the oxygen reduction reaction. *Sci. Rep*. **5**, 17385; doi: 10.1038/srep17385 (2015).
